# Therapeutic Potential of Ambroxol in Osteoarthritis: A Drug Repurposing Study

**DOI:** 10.3390/ph19050677

**Published:** 2026-04-27

**Authors:** Michelline Joana Tenório Albuquerque Madruga Mesquita, Anne Caroline Silva Nogueira da Cruz, Joana Tenório Albuquerque Madruga Mesquita Meireles Teixeira, Amanda Tissore Forwille Reis, Gustavo Medeiro Frota, Taciana Gabrielle Pinheiro de Moura Carvalho, Rafael Antônio Freire Carvalho, Jorge Antônio Meireles Teixeira, Marcelo Souza de Andrade, Rafael Cardoso Carvalho, Eduardo Martins de Sousa, Joicy Cortez de Sá Sousa, Sebastião Vieira de Morais, Eduardo Rodrigues Silva, Maria do Socorro de Sousa Cartágenes, João Batista Santos Garcia

**Affiliations:** 1 Post-Graduate Program in Health Sciences (UFMA), Medicine Departament I, Federal University of Maranhao (UFMA), Sao Luis 65080-805, MA, Brazil; 2 Post-Graduate Program in Health Sciences, Federal University of Maranhao (UFMA), Sao Luis 65080-805, MA, Brazil; anne_caroliness@hotmail.com; 3 Medical School, University CEUMA, Sao Luis 65075-120, MA, Brazil; tenoriojoana04@gmail.com; 4 College of Pharmacy, Federal University of Maranhao (UFMA), Sao Luis 65080-805, MA, Brazil; amanda.forwille@discente.ufma.br; 5 Graduate Program in Biotechnology—RENORBIO, Federal University of Maranhao (UFMA), Sao Luis 65080-805, MA, Brazil; gustavo.frota@discente.ufma.br (G.M.F.); eduardo.rs@discente.ufma.br (E.R.S.); 6 Medicine Department I, Federal University of Maranhao (UFMA), Sao Luis 65080-805, MA, Brazil; taciana.moura@ufma.br (T.G.P.d.M.C.); jorge.meireles@ufma.br (J.A.M.T.); 7 Medical School, Pinheiro Campus, Federal University of Maranhao (UFMA), Pinheiro 65200-000, MA, Brazil; rafael.afc@ufma.br; 8 Laboratory of Molecular Biology (Labiomol), Post Graduation Program in Adult Health (PPGSAD), Federal University of Maranhao (UFMA), Sao Luis 65080-805, MA, Brazil; marcelo.andrade@ufma.br; 9 Special Coordination in Biological and Health Sciences I, Center for Biological and Health Sciences (CCBS) Federal University of Maranhao (UFMA), Sao Luis 65080-805, MA, Brazil; carvalho.rafael@ufma.br; 10 Graduate Program in Applied Biosciences to Health, University CEUMA, Sao Luis 65075-120, MA, Brazil; eduardo.martins@ceuma.br; 11 Special Coordination of Morphology (CEMOR/CCBS/UFMA), Graduate Program in Health Sciences—PPGCS, Federal University of Maranhao (UFMA), Sao Luis 65080-805, MA, Brazil; joicy.sa@ufma.br; 12 Medicine Department II, Federal University of Maranhao (UFMA), Sao Luis 65080-805, MA, Brazil; svmcoluna@gmail.com (S.V.d.M.); joao.garcia@ufma.br (J.B.S.G.); 13 Physiological Sciences Department, Federal University of Maranhao (UFMA), Sao Luis 65080-805, MA, Brazil; cartagenes.maria@ufma.br

**Keywords:** ambroxol, drug repurposing, experimental osteoarthritis, inflammation, chronic pain

## Abstract

**Background/Objectives**: Ambroxol is a mucolytic agent widely used in the treatment of respiratory diseases; however, evidence in the literature indicates anti-inflammatory, analgesic, and immunomodulatory properties, suggesting potential for therapeutic repositioning. This study aimed to evaluate the analgesic and anti-inflammatory effects of ambroxol in an experimental model of osteoarthritis (OA). **Methods**: Adult male Wistar rats underwent OA induction on day zero (D0) by sodium monoiodoacetate (MIA) injection and were allocated into the following groups: Healthy, negative control (CTRL−), and groups treated with meloxicam (2 mg/kg) or ambroxol (10, 50, and 100 mg/kg). Treatments were administered orally (gavage) once daily for 28 days. Behavioral tests were performed, including rotarod, walkway gait analysis, weight-bearing, Von Frey, and Rat Grimace Scale assessments, along with radiographic and histopathological analyses and quantification of pro- and anti-inflammatory cytokines (IL-1β, IL-6, and IL-10). **Results**: Ambroxol treatment improved nociceptive parameters and motor function, reduced radiographic and histopathological scores, and showed performance comparable to meloxicam in several tests. There was a marked reduction in IL-1β and IL-6 levels, while IL-10 levels were lower in ambroxol-treated groups, suggesting early control of the inflammatory response. **Conclusions**: The results indicate that ambroxol exhibits antinociceptive and anti-inflammatory actions and suggest a potential chondroprotective effect, reinforcing its viability as a candidate for therapeutic repositioning in osteoarthritis. Further studies are required to more precisely elucidate its mechanisms of action, define optimal dosing and treatment duration, and support translation to clinical models.

## 1. Introduction

Osteoarthritis (OA) is a multifactorial joint disease resulting from an imbalance between the processes of cartilage degradation and regeneration, influenced by biomechanical, inflammatory, genetic, and metabolic factors. It is characterized by the progressive degeneration of articular cartilage, remodeling of the subchondral bone, inflammation of the synovial membrane, and alterations in periarticular tissues. Among these structures, articular cartilage is the most affected, playing a central role in the pathophysiology of the disease [1,2].

The initial event of cartilage degradation has not yet been completely established. However, it is known that articular cartilage has a low regenerative capacity because it is avascular, aneural, and alymphatic. In this context, repetitive mechanical overload or joint trauma may induce microlesions in the subchondral bone, promoting joint instability. Tissue aging also contributes to the establishment of cellular senescence, characterized by the permanent interruption of the cell cycle of chondrocytes and synoviocytes. These cells begin to maintain a metabolically active state, secreting inflammatory mediators and degradative enzymes that compromise the integrity of the extracellular matrix and favor the progression of the articular degenerative process [1,2,3].

The progression of osteoarthritis (OA) is strongly mediated by inflammatory processes, in which pro-inflammatory cytokines, particularly interleukin-1 beta (IL-1β), tumor necrosis factor alpha (TNF-α), and interleukin-6 (IL-6), produced by chondrocytes and synoviocytes activated in response to cartilage and subchondral bone injury, stimulate the production of proteolytic enzymes, such as matrix metalloproteinases (MMPs) and aggrecanases of the ADAMTs family. These enzymes are responsible for the degradation of structural components of cartilage, including type II collagen and aggrecan, directly contributing to the deterioration of the extracellular matrix [1,4].

Concomitantly, there is an increase in oxidative stress with the production of reactive oxygen species and nitric oxide, as well as activation of the arachidonic acid pathway and cyclooxygenase-2 (COX-2), culminating in the production of prostaglandin E2 (PGE2), a mediator associated with inflammation, joint pain, and the progression of cartilage degradation [5,6,7].

In response to this inflammatory environment, anti-inflammatory cytokines, such as interleukin-10 (IL-10), are produced as a compensatory mechanism, acting to inhibit the production of pro-inflammatory cytokines and to reduce the expression of matrix-degrading enzymes. However, in osteoarthritis, these regulatory mechanisms are generally insufficient to neutralize the intense catabolic activity present in the articular microenvironment, thereby favoring the structural progression of the disease [5].

Given this complex inflammatory and degenerative environment, the treatment of osteoarthritis primarily aims to control pain, reduce low-grade inflammation, and prevent the structural progression of the disease. To achieve these goals, both non-pharmacological and pharmacological interventions are used, including analgesics, nonsteroidal anti-inflammatory drugs (NSAIDs), corticosteroids, and hyaluronic acid. Among these options, NSAIDs and selective inhibitors of the cyclooxygenase-2 (COX-2) enzyme are the most frequently employed due to their action in reducing inflammatory prostaglandins. However, these medications are often associated with significant adverse effects, including cardiovascular and gastrointestinal complications [8,9].

The therapeutic limitations and safety profile of these approaches reinforce the need for safer and more effective pharmacological strategies for the management of osteoarthritis. In this context, drug repurposing has emerged as a promising strategy, as it seeks new applications for already approved medications, thereby reducing costs and development time [10,11].

Ambroxol (trans-4-(2-amino-3,5-dibromobenzylamino)-cyclohexanol), an active metabolite of bromhexine, has attracted increasing scientific interest due to its broad pharmacological spectrum. Experimental and clinical evidence demonstrate that this drug exhibits anti-inflammatory, local analgesic, antioxidant, and antimicrobial properties, in addition to stimulating the production of pulmonary surfactant. Additionally, ambroxol presents a favorable safety profile, even when administered at high doses [12,13].

Studies demonstrate that ambroxol can reduce the production of important pro-inflammatory cytokines, such as TNF-α, IL-1β, and IL-6, in addition to increasing the expression of anti-inflammatory cytokines, such as interleukin-10 (IL-10), suggesting a relevant role in the regulation of the inflammatory response [14,15,16,17]. The reduction in these pro-inflammatory cytokines may also indirectly influence cartilage metabolism, since mediators such as IL-1β and IL-6 stimulate the expression of matrix metalloproteinases (MMPs) and aggrecanases, enzymes directly involved in the degradation of structural components of cartilage [4,18,19,20].

Several experimental studies have investigated the anti-inflammatory potential of ambroxol in different models of inflammatory and immune-mediated diseases. In an experimental model of cyclophosphamide-induced cystitis, treatment with ambroxol significantly reduced TNF-α levels and promoted improvement in the histopathological parameters of the tissue. Similar results were observed in models of psoriasis-like cutaneous inflammation and in models of inflammatory colitis, in which ambroxol was able to reduce inflammatory cytokines, decrease oxidative stress, and attenuate tissue damage. These findings reinforce the concept that ambroxol may act as a multifunctional immunomodulatory agent capable of modulating inflammatory cascades and oxidative processes in different pathological contexts [17,21,22].

Considering that osteoarthritis involves a complex interaction between chronic inflammation, oxidative stress, and progressive degradation of articular cartilage, drugs capable of modulating these mechanisms represent promising candidates for therapeutic repurposing strategies. In this context, the anti-inflammatory, antioxidant, and immunomodulatory properties of ambroxol provide a plausible mechanistic rationale for investigating its effects in osteoarthritis. Thus, the present study investigated the anti-inflammatory and antinociceptive potential of ambroxol in an experimental model of osteoarthritis induced by sodium monoiodoacetate (MIA) in rat knees.

## 2. Results

### 2.1. Behavioral and Functional Assessments

**Figure 1:**
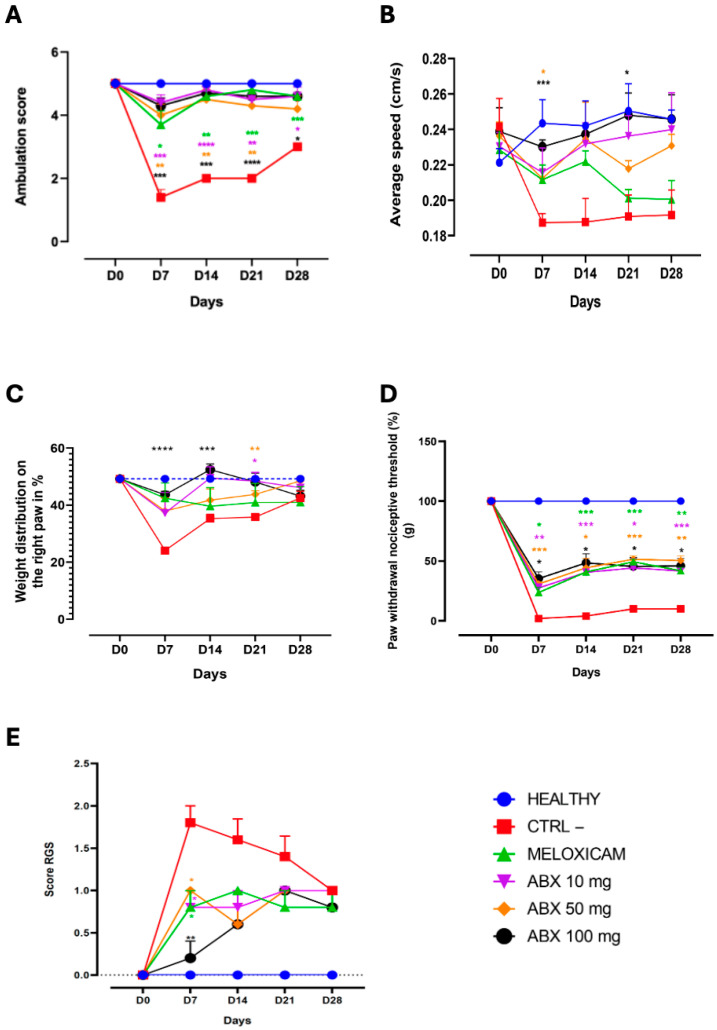
Behavioral and nociceptive assessment in rats with MIA-induced osteoarthritis treated with ambroxol (10, 50, and 100 mg/kg) or meloxicam. (**A**) Rotarod test for evaluation of motor activity and coordination; (**B**) gait analysis to assess mean locomotion velocity; (**C**) weight-bearing test for measurement of spontaneous postural pain; (**D**) mechanical paw withdrawal threshold assessed using the Von Frey test; (**E**) spontaneous pain evaluation using the Rat Grimace Scale (RGS). Assessments were performed on days 0, 7, 14, 21, and 28 after osteoarthritis induction with monoiodoacetate (MIA). Animals treated with ambroxol showed progressive improvement in locomotor parameters and a reduction in behavioral pain indicators compared with the negative control group (CTRL−), suggesting an analgesic effect and attenuation of functional impairment associated with the experimental osteoarthritis model. Data are expressed as mean ± SEM, with *n* = 5 animals per group. Symbols indicate statistically significant differences compared with the CTRL− group (* p < 0.05, ** *p* < 0.01, *** *p* < 0.001, and **** *p* < 0.0001), with colors matching the respective groups. Statistical comparisons were performed using two-way repeated-measures ANOVA followed by Tukey’s post hoc test.

Motor performance, assessed by the rotarod test, showed a reduction in motor activity in the negative control group (CTRL−) at D7 following osteoarthritis induction. In contrast, animals treated with ambroxol at doses of 10, 50, and 100 mg/kg exhibited a progressive recovery of locomotor function, with the 10 mg/kg and 100 mg/kg doses showing more pronounced effects that were maintained up to D28 compared with the CTRL− group. The response profile of ambroxol was comparable to that observed with meloxicam (Figure 1A).

In the spontaneous gait analysis on the walkway, the CTRL− group showed a reduction in mean walking speed from D7 onward, indicating functional impairment. In contrast, ambroxol-treated groups maintained higher mean velocities than the CTRL− group at all evaluated time points, with the 50 mg/kg and 100 mg/kg doses showing statistically significant differences at D7. Notably, the 100 mg/kg dose maintained statistical significance at D21, suggesting preservation of motor performance (Figure 1B).

In the incapacitance (weight-bearing) test, the CTRL− group showed a reduction in weight distribution on the affected paw at D7, confirming increased pain. Ambroxol treatment promoted a dose- and time-dependent functional recovery. The 100 mg/kg dose induced a faster and more pronounced recovery from D7 onward, maintaining a statistically significant difference compared with the CTRL− group up to D14. The 50 mg/kg and 10 mg/kg doses showed statistically significant differences at D21 (Figure 1C).

Mechanical allodynia assessed by the von Frey test demonstrated that ambroxol increased the nociceptive threshold compared with the CTRL− group from D7 onward. Doses of 10, 50, and 100 mg/kg produced early and sustained antinociceptive effects comparable to those observed with meloxicam, indicating a consistent and long-lasting analgesic effect (Figure 1D).

Spontaneous pain, assessed by the Rat Grimace Scale (RGS), showed a marked increase in scores in the CTRL− group following OA induction at D7. All ambroxol-treated groups, as well as the meloxicam group, exhibited a significant reduction in facial pain scores at D7, with ambroxol-treated animals displaying facial expressions comparable to those observed in the meloxicam-treated group, which was the reference drug. The most pronounced effect was observed at the 100 mg/kg dose. At D14 and D21, ambroxol- and meloxicam-treated groups maintained low RGS values, indicating sustained antinociceptive effects over time (Figure 1E).

### 2.2. Radiographic and Histological Evaluation

**Figure 2:**
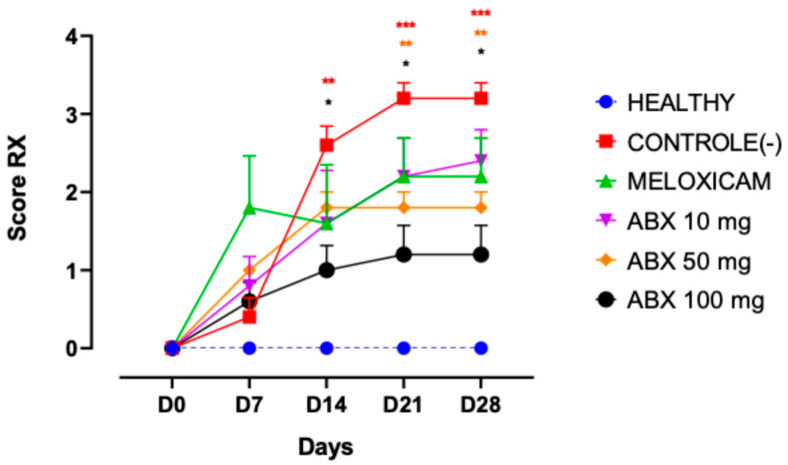
Radiographic evaluation of osteoarthritis progression in rats subjected to sodium monoiodoacetate (MIA)-induced osteoarthritis and treated with ambroxol (10, 50, and 100 mg/kg) or meloxicam. The degree of joint damage was assessed using the Kellgren–Lawrence (KL) radiographic scoring system in the right knee on days 0, 7, 14, 21, and 28 after osteoarthritis induction. Animals treated with ambroxol exhibited lower radiographic scores over time compared with the negative control group (CTRL−), indicating attenuation of structural joint deterioration associated with the experimental osteoarthritis model. Data are expressed as mean ± SEM, with *n* = 5 animals per group. Statistical comparisons were performed using two-way repeated-measures ANOVA followed by Tukey’s post hoc test. Statistically significant differences compared with the CTRL− group are indicated by * *p* < 0.05, ** *p* < 0.01, and *** *p* < 0.001. The colors of the asterisks correspond to the respective experimental groups.

Radiographic and histological analyses revealed a consistent pattern of joint degeneration in animals from the CTRL− group and a protective effect in groups treated with ambroxol and meloxicam. Radiographic evaluation demonstrated that ambroxol exerted a dose-dependent effect. The group treated with ambroxol at 100 mg/kg showed a significant reduction in Kellgren–Lawrence (KL) scores compared with the CTRL− group from D14 onward, maintaining sustained improvement up to D28, indicating an effect on limiting osteoarthritis progression. The 50 mg/kg dose significantly reduced KL scores compared with the CTRL− group at D21 and D28, exhibiting a radiographic pattern similar to that observed with meloxicam, thus demonstrating efficacy, although with a later onset of response compared with the higher ambroxol dose (Figure 2).

**Figure 3:**
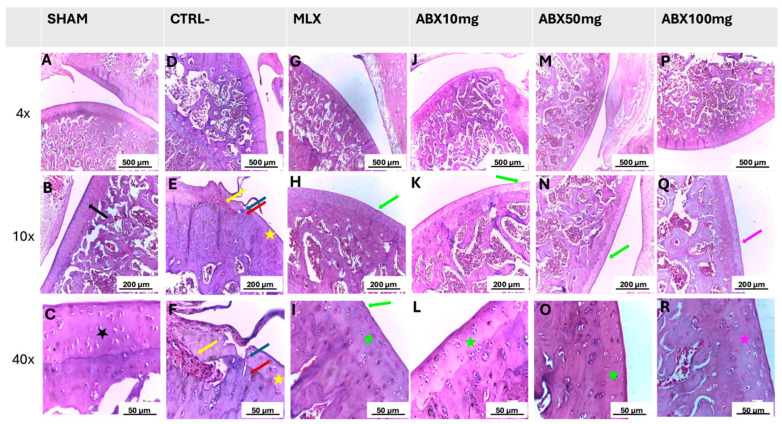
Histological evaluation of knee articular cartilage in rats subjected to the experimental model of osteoarthritis induced by sodium monoiodoacetate (MIA). Composite panel of representative images from the following groups: HEALTHY (**A**–**C**), negative control (CTRL−; **D**–**F**), meloxicam (MLX; **G**–**I**), ambroxol 10 mg/kg (ABX10; **J**–**L**), ambroxol 50 mg/kg (ABX50; **M**–**O**), and ambroxol 100 mg/kg (ABX100; **P**–**R**). The samples were stained with hematoxylin and eosin (H&E) and analyzed at magnifications of 4×, 10×, and 40×, with corresponding scale bars of 500 μm (4×), 200 μm (10×), and 50 μm (40×). In animals from the HEALTHY group (**A**–**C**), preservation of cartilage architecture is observed, with intact cartilage [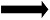] and organized chondrocytes [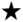]. In contrast, the CTRL− group (**D**–**F**) exhibits degenerative alterations consistent with advanced osteoarthritis, including fissuring in the deep cartilage layer [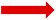], fragmented Tidemark [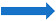], chondrocyte depletion [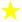], and the presence of osteoprogenitor cells and osteoclasts [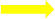]. The groups treated with MLX (**G**–**I**), ABX 10 mg/kg (**J**–**L**), and ABX 50 mg/kg (**M**–**O**) show mild areas of superficial fibrillation [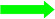] and intact cells [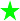], suggesting attenuation of the degenerative changes induced by MIA. In the group treated with ABX 100 mg/kg (**P**–**R**), an almost intact articular surface is observed, with rare fibrillations, preserved tidemark [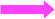], and intact cells [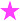], indicating greater preservation of the articular cartilage compared with the negative control group (CTRL−).

The histological analysis corroborated the radiographic findings, revealing marked differences in cartilage architecture among the experimental groups. The healthy group preserved intact cartilage structure with well-organized chondrocytes (Figure 3A–C), whereas the negative control group (CTRL−) exhibited extensive chondral lesions, fissuring in the deep cartilage layer, fragmented tidemark, chondrocyte depletion, and the presence of osteoprogenitor cells and osteoclasts (Figure 3D–F). The meloxicam and ambroxol 10 and 50 mg/kg groups (Figure 3G–O) showed superficial fibrillation with preservation of cellularity. In contrast, the ambroxol 100 mg/kg group displayed cartilage morphology similar to that observed in the healthy group, with only rare fibrillations (Figure 3P–R).

**Figure 4:**
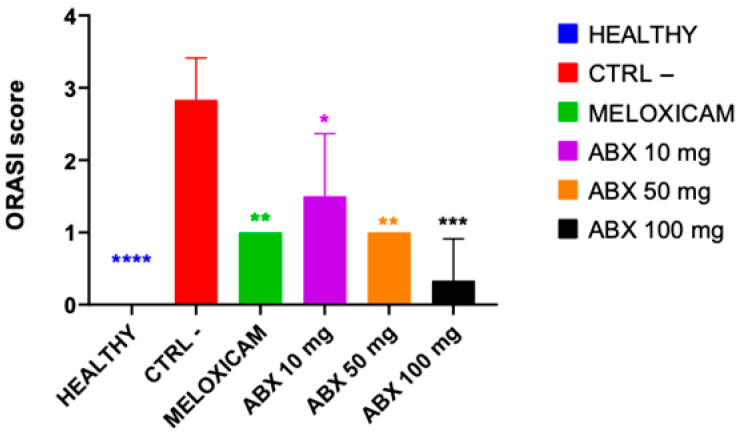
Histological evaluation of articular cartilage using the Osteoarthritis Research Society International (OARSI) scoring system in rats subjected to the experimental model of osteoarthritis induced by sodium monoiodoacetate (MIA). The scores reflect the degree of articular cartilage degeneration in the different experimental groups: HEALTHY, negative control (CTRL−), meloxicam (MLX), ambroxol 10 mg/kg (ABX10 mg), ambroxol 50 mg/kg (ABX50 mg), and ambroxol 100 mg/kg (ABX100 mg). The CTRL− group showed a significant increase in the OARSI score, indicating a greater degree of cartilage damage following osteoarthritis induction. In contrast, the groups treated with meloxicam and ambroxol exhibited reduced histological scores, suggesting attenuation of degenerative cartilage changes. This effect was more evident in the groups treated with ambroxol at intermediate and higher doses, indicating a potential chondroprotective effect of the drug in the experimental osteoarthritis model. Data are expressed as mean ± SEM, with *n* = 5 animals per group. Statistical analysis was performed using one-way ANOVA followed by Tukey’s post hoc test. Statistical differences are indicated by * *p* < 0.05, ** *p* < 0.01, *** *p* < 0.001, and **** *p* < 0.0001 compared with the CTRL− group. The colors of the asterisks indicate the respective groups with statistically significant differences.

The mean Osteoarthritis Research Society International (OARSI) scores were 1.5 ± 0.5 for ambroxol 10 mg/kg; 1.0 ± 0.0 for meloxicam and ambroxol 50 mg/kg; and 0.33 ± 0.33 for ambroxol 100 mg/kg, whereas the CTRL− group exhibited the highest values (2.83 ± 0.33). These results further support that ambroxol promoted structural preservation of articular cartilage comparable to that observed with meloxicam (Figure 4).

### 2.3. Cytokine Quantification

**Figure 5:**
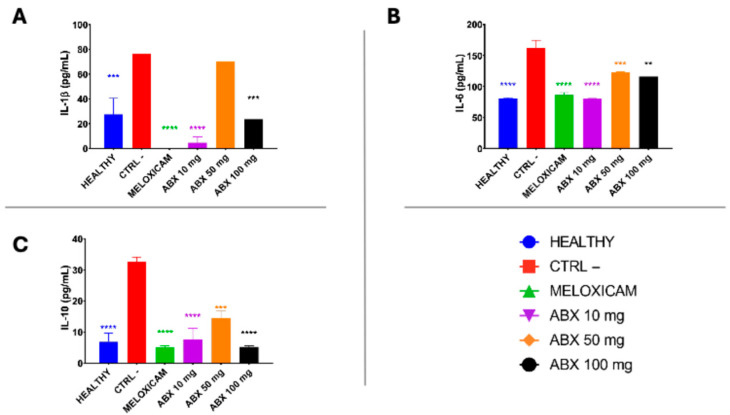
Evaluation of serum levels of systemic inflammatory cytokines in rats subjected to the experimental model of osteoarthritis induced by sodium monoiodoacetate (MIA). (**A**) IL-1β; (**B**) IL-6; (**C**) IL-10. Serum cytokine concentrations were determined by ELISA and expressed in pg/mL in the different experimental groups: HEALTHY, negative control (CTRL−), meloxicam (MLX), ambroxol 10 mg/kg (ABX10 mg), ambroxol 50 mg/kg (ABX50 mg), and ambroxol 100 mg/kg (ABX100 mg). The CTRL− group showed a significant increase in the pro-inflammatory cytokines IL-1β and IL-6, reflecting the systemic inflammatory state associated with experimental osteoarthritis. In contrast, the groups treated with meloxicam and ambroxol demonstrated a significant reduction in these inflammatory mediators. In addition, a relative increase in the anti-inflammatory cytokine IL-10 was observed, suggesting modulation of the systemic inflammatory profile by the treatment. Data are presented as mean ± SEM, *n* = 5 animals per group. Statistical analysis was performed using one-way ANOVA followed by Tukey’s post hoc test. Statistical differences are indicated by ** *p* < 0.01, *** *p* < 0.001, and **** *p* < 0.0001 compared with the CTRL− group. The colors of the asterisks indicate the respective groups with statistically significant differences.

Cytokine quantification showed that the CTRL− group exhibited elevated levels of IL-1β and IL-6 compared with the other groups, indicating an intense systemic inflammatory process. In contrast, animals in the healthy group displayed significantly lower concentrations of these cytokines, confirming the absence of an inflammatory response (Figure 5A,B).

Treatment with meloxicam and ambroxol at doses of 10 and 100 mg/kg promoted a significant reduction in IL-1β levels compared with the CTRL− group. Regarding IL-6, all treatments significantly reduced serum concentrations when compared with the CTRL− group (Figure 5A,B).

The anti-inflammatory cytokine IL-10 showed significantly lower levels in animals treated with meloxicam and ambroxol at doses of 10, 50, and 100 mg/kg compared with the CTRL− group (Figure 5C), indicating modulation of the immune response by the evaluated treatments.

## 3. Discussion

The present study demonstrated that ambroxol exerted significant analgesic and anti-inflammatory effects in an experimental model of osteoarthritis (OA) induced by sodium monoiodoacetate (MIA) in rats, in addition to preserving joint integrity. Behavioral assessments revealed a reduction in spontaneous pain and mechanical allodynia, as well as improvements in locomotor performance and weight distribution between the hind paws. Taken together, radiographic, histological, and immunological findings indicated that ambroxol was able to preserve articular structure and modulate the expression of pro- and anti-inflammatory cytokines, exhibiting efficacy comparable to that of the reference drug meloxicam.

All results obtained were analyzed in comparison with the negative control group (CTRL−). In the functional assessments performed using the rotarod, weight-bearing, and gait analysis tests, progression of osteoarthritis was observed in the negative control group (CTRL−), characterized by reduced locomotor performance, decreased weight support on the affected limb, and a reduction in mean walking speed starting from day 7. These findings reflect functional impairment resulting from pain and joint degeneration, parameters widely recognized as markers of OA progression [23,24,25].

However, on day 28, a slight increase in weight-bearing values was observed in the CTRL− group, approaching those of the other groups. This finding may reflect spontaneous compensatory mechanisms typical of chronic pain models, in which animals, even in the presence of persistent pain, tend to develop adaptive weight redistribution strategies over time. This phenomenon has been widely documented in the literature as part of the behavioral adjustments associated with the progression of osteoarthritis [26].

In this study, locomotor performance assessed by the rotarod test was significantly improved in the groups treated with ambroxol. Complementarily, gait analysis demonstrated that treatment with ambroxol at doses of 50 and 100 mg/kg significantly increased walking speed. These findings indicate an improvement in locomotor function, possibly related to the reduction in pain and joint inflammation, factors that play a central role in the functional limitation observed in osteoarthritis. In this context, the analgesic and anti-inflammatory effects previously described for ambroxol may contribute to the preservation of mobility in experimental models of the disease [12,27].

Similar results have been reported in the literature. In a rat model of Parkinson’s disease, ambroxol improved motor coordination and locomotor performance, reinforcing its potential modulatory effect on mechanisms associated with motor dysfunction [28].

Corroborating these findings, the weight-bearing test demonstrated a significant improvement in the ability of the affected paw to support weight in the groups treated with ambroxol. The 100 mg/kg dose showed an early and sustained effect, with values approaching those observed in the healthy group. These results are consistent with previous studies describing the ability of ambroxol to effectively suppress pain symptoms in animal models of chronic, inflammatory, and neuropathic pain [12,27].

Considering that functional recovery is directly associated with pain modulation, nociceptive tests using the Von Frey test and the Rat Grimace Scale (RGS) were performed. In the Von Frey test, the groups treated with ambroxol exhibited a progressive and significant increase in the response threshold from the beginning of treatment, indicating an anti-allodynic effect. Similar results have been previously reported in the literature. In a model of chronic inflammatory pain in rats, ambroxol reduced mechanical allodynia and hyperalgesia [28]. In that study, the Von Frey test was also used, in which treated animals showed a significant decrease in nociceptive responses, corroborating the antinociceptive potential of ambroxol.

The results obtained using the Rat Grimace Scale (RGS) revealed a modulation of spontaneous pain by ambroxol. The treated groups showed a reduction in RGS scores over the same period, with significant differences observed at doses of 10, 50, and 100 mg/kg on day 7, displaying a pattern similar to that observed with meloxicam. By day 28, all groups exhibited similar scores, which may be attributed to adaptive neurobehavioral mechanisms and facial desensitization frequently described in chronic pain models [29].

These effects may be related to the ability of ambroxol to inhibit voltage-gated sodium channels, particularly Nav1.8, which are widely expressed in peripheral nociceptive neurons, as well as possible effects on Nav1.9, as previously described in the literature. The modulation of these channels reduces neuronal excitability and peripheral nociceptive transmission, contributing to the reduction in hyperalgesia and mechanical allodynia observed in this study [28,30].

The integration of behavioral outcomes with morphofunctional analyses broadens the understanding of the pathophysiological mechanisms underlying the experimental pain model and the therapeutic effects of ambroxol. The behavioral tests employed in this study allowed the characterization of both nociceptive and spontaneous pain. Importantly, to the best of our knowledge, the use of walkway gait analysis, the weight-bearing test, and the Rat Grimace Scale (RGS) to evaluate ambroxol in an experimental pain model has not been previously reported in the literature.

Radiographic analysis based on the Kellgren–Lawrence scale demonstrated the progression of joint degeneration throughout the experimental protocol, with the CTRL− group showing a significant increase in scores from day 14 onward, confirming the effectiveness of the MIA-induced osteoarthritis model. In contrast, the groups treated with ambroxol exhibited a delay in the progression of structural changes, particularly at the 100 mg/kg dose, which showed statistically significant differences from day 14 and remained so until day 28, and at the 50 mg/kg dose, which exhibited significantly lower scores from day 21 through to day 28, with performance comparable to that observed with meloxicam.

Radiographic findings suggest that ambroxol may be associated with a delay in the progression of structural articular changes in treated groups; however, to the best of our knowledge, there is no evidence in the literature directly linking its use to radiographic outcomes in osteoarthritis models, meaning that this application is still poorly explored. These effects may be related to previously described pharmacological mechanisms, particularly its anti-inflammatory action mediated by the inhibition of pro-inflammatory cytokines such as IL-1β, IL-6, and TNF-α, as well as its antioxidant properties through the neutralization of reactive oxygen species (ROS), which reduce oxidative stress, preserve the extracellular matrix, and promote chondrocyte viability, thereby contributing to the attenuation of osteoarthritis progression [21,31,32].

Histological evaluation corroborated the radiographic findings. The CTRL− group exhibited high scores, characterized by marked cartilage degeneration, extracellular matrix disorganization, and loss of chondral architecture. In contrast, ambroxol-treated groups showed a significant reduction in histological scores, indicating preservation of cartilage tissue. The 100 mg/kg dose showed the best performance, whereas the 50 mg/kg dose demonstrated an effect comparable to that of the standard anti-inflammatory agent. The 10 mg/kg dose, although significant compared with the negative control, showed lower efficacy, suggesting a dose-dependent effect. This pattern is supported by previous studies demonstrating greater efficacy of ambroxol at higher doses in controlling inflammation and pain [21,28].

The cytokine analysis revealed the anti-inflammatory profile of ambroxol. Cytokines were quantified in the serum of the animals, allowing a systemic evaluation of the inflammatory response associated with the experimental osteoarthritis model. Although osteoarthritis is predominantly a disease localized to the joint, inflammatory mediators produced in the articular microenvironment can reach the systemic circulation and reflect the inflammatory activity associated with joint degeneration [18,33].

In this context, the CTRL− control group exhibited elevated levels of IL-1β and IL-6, consistent with a pronounced inflammatory process, whereas the treated groups showed significant reductions in these mediators, particularly IL-1β at doses of 10 and 100 mg/kg and IL-6 at all tested doses, with effects comparable to those observed with meloxicam. These results reinforce the ability of ambroxol to modulate joint inflammation through the inhibition of key pro-inflammatory cytokines involved in cartilage degradation, such as IL-1β and IL-6, in agreement with previous evidence reported in the literature [16,21,32,34].

Regarding IL-10, an anti-inflammatory cytokine, it was observed that the CTRL− group exhibited the highest concentrations, whereas the groups treated with meloxicam and ambroxol showed significantly lower levels, similar to those observed in the healthy group. The lower production of this cytokine in the treated groups may reflect a reduction in the primary inflammatory stimulus, since IL-10, despite its anti-inflammatory role, may also act as a marker of the intensity of the inflammatory response under established pathological conditions. Thus, elevated IL-10 levels in the untreated group may represent a compensatory mechanism in response to exacerbated inflammation, whereas the reduction observed in the treated groups may indicate better control of the inflammatory process, with a reduced need for activation of immunoregulatory mechanisms [35]

Additionally, a previous study investigating a model of severe pediatric pneumonia demonstrated that elevated IL-10 levels were associated with greater inflammatory severity and that their reduction following treatment with ambroxol corresponded to a marker of clinical improvement [35]. Although this model represents a distinct pathological context, these findings suggest that a decrease in IL-10 may occur in scenarios in which attenuation of the primary inflammatory stimulus reduces the need for compensatory anti-inflammatory responses.

The integration of radiographic, histological, and immunological findings suggests that the chondroprotective effects of ambroxol are largely attributable to its anti-inflammatory and immunomodulatory actions on the articular microenvironment. The reduction in IL-1β and IL-6 in the treated groups reflects the suppression of pro-inflammatory mediators directly involved in cartilage degradation and in the activation of chondrocytes and osteoclasts, in agreement with the lower radiographic and histological scores, which indicate preservation of chondral architecture. Although the present study did not directly evaluate the tissue distribution of the drug within the joint, it is plausible that the systemic modulation of these inflammatory mediators contributes to the reduction in the catabolic environment associated with cartilage degradation.

Considering the translational potential of these findings, the interpretation of the doses used in this study should be analyzed within the context of interspecies dose equivalence. The ambroxol doses were defined based on previously published preclinical data and on calculations of animal equivalent dose (AED), using allometric scaling recommended by the guidelines of the U.S. Food and Drug Administration [36,37]. According to these principles, the maximum dose used in this study (100 mg/kg/day) corresponds approximately to a human dose considered safe of up to 1000 mg/day. Previous clinical studies have demonstrated that ambroxol presents a favorable safety profile even under high-dose regimens, suggesting that the pharmacological exposure observed in this experimental model may be plausible from a translational perspective [28,38]. However, further pharmacokinetic investigations and additional clinical studies are necessary to determine the therapeutic feasibility of this strategy in the context of osteoarthritis.

Despite the promising results observed in this study, some limitations should be considered. The absence of the evaluation of other relevant cytokines, such as TNF-α, and mediators, including NF-κB, MMP-13, and ADAMTS-5, represents a limitation of the study and highlights the need for future investigations that more comprehensively explore the systemic and local inflammatory profile associated with ambroxol treatment.

Another limitation refers to the absence of characterization of immune cells present in the articular tissue. Although the results indicate significant modulation of inflammatory cytokines associated with ambroxol treatment, specific analyses were not performed to identify the cellular sources of these mediators within the articular microenvironment.

Therefore, the results of this study contribute scientifically by demonstrating, in an integrated manner, the multimodal potential of ambroxol as an anti-inflammatory, analgesic, and chondroprotective agent in an experimental model of osteoarthritis. The convergence of behavioral, radiographic, histological, and immunological findings strengthens the relevance of the study and broadens the understanding of the mechanisms of action of ambroxol in the modulation of pain and the preservation of articular cartilage. These findings support the repositioning of ambroxol as a promising therapeutic alternative and open perspectives for translational investigations aimed at the management of chronic pain and osteoarticular degeneration.

## 4. Materials and Methods

### 4.1. Drugs

The drugs used in this study were commercially acquired. Ambroxol was stored at room temperature until use and administered by oral gavage at doses of 10, 50, and 100 mg/kg/day. Meloxicam was used as a pharmacological control and administered by oral gavage at a dose of 2 mg/kg/day.

### 4.2. Animals

Adult male Wistar rats (*Rattus norvegicus*, albinus variety), approximately 120 days old and weighing 300 ± 50 g, were used in this study. The animals were obtained from the Central Animal Facility of the Federal University of Maranhão (UFMA) and housed in the Sectorial Animal Facility of the Center for Biological and Health Sciences at UFMA under controlled conditions of temperature (22 ± 2 °C), humidity (40–60%), and a 12 h light/dark cycle, with free access to commercial chow (Nuvilab^®^, Quimtia, Colombo, PR, Brazil) and water ad libitum. Animals underwent a seven-day acclimatization period for clinical monitoring and environmental adaptation and were subsequently randomized and blindly allocated to the experimental groups.

### 4.3. Ethical Procedure

The study was conducted at the Experimental Laboratory for the Study of Pain (LEED), affiliated with the Federal University of Maranhão (UFMA). The experimental protocol was previously approved by the Institutional Animal Care and Use Committee of UFMA (CEUA/UFMA) under protocol number 23115.013021/2022-17. All experimental procedures were conducted in accordance with the ethical guidelines for animal experimentation established by the Brazilian National Council for the Control of Animal Experimentation (CONCEA) and the International Association for the Study of Pain [39,40].

### 4.4. Experimental Protocol

The study included male Wistar rats (*n* = 30), randomly allocated into six experimental groups (A–F), with five animals per group (*n* = 5/group). On day 0 (D0), all animals underwent baseline behavioral assessments and radiographic examinations of the right knee. Osteoarthritis was induced by a single intra-articular injection of 2 mg sodium monoiodoacetate (MIA) [24]. The procedure was performed under general anesthesia induced via intraperitoneal administration of ketamine hydrochloride (50 mg/kg), midazolam (1 mg/kg), and tramadol (5 mg/kg), with anesthetic maintenance using 2.5% isoflurane. After the procedure, animals were housed in individual cages under a 12 h light/dark cycle, with free access to food and water, throughout the 28-day experimental protocol.

Treatment was initiated on the third day after osteoarthritis induction and administered once daily by oral gavage. Group A (healthy) remained without induction or treatment; Group B, the negative control (CTRL−), received 0.9% saline (0.1 mL/kg/day); Group C was treated with meloxicam (MLX, 2 mg/kg/day); and Groups D, E, and F received ambroxol (ABX) at doses of 10, 50, and 100 mg/kg/day, respectively. Behavioral and radiographic evaluations were performed on days 0, 7, 14, 21, and 28. On day 28, after completion of the analyses, animals were euthanized for collection of right knee joints and blood samples for histopathological analyses and cytokine quantification.

The randomization of animals was performed using a computer-generated allocation sequence, and the distribution into experimental groups was conducted by a researcher who was not involved in the subsequent outcome assessments. Behavioral, radiographic, and histological evaluations were performed by previously trained evaluators who were blinded to the allocation of animals to the experimental groups, in order to minimize potential observation bias. For statistical analysis, the datasets were previously coded so that the researcher responsible for the analysis remained blinded to the identification of the experimental groups during data processing.

### 4.5. Parameters for Determination of Administered Doses

The ambroxol doses used (10, 50, and 100 mg/kg/day) were defined based on previously published preclinical data and on the calculation of the Animal Equivalent Dose (AED), following the principles of allometric scaling recommended by the guidelines of the U.S. Food and Drug Administration [36,37].

Previous studies have demonstrated a linear correlation between plasma ambroxol levels in humans and rodents, suggesting a wide safety margin for dose extrapolation. In addition, clinical data report the safe use of doses of up to 1000 mg/day in humans, further supporting the feasibility of the dose ranges tested [28,38].

For this study, AED calculations considered a 60 kg human and a 150 g rat. The meloxicam dose (2 mg/kg/day) corresponds to the therapeutic human dose of 15 mg/day [41]. The lowest ambroxol dose (10 mg/kg/day) was derived from the standard clinical regimen of 90 mg/day [42]. The intermediate dose (50 mg/kg/day) was defined based on the Effective Dose 50% (ED50), from literature data demonstrating its association with significant anti-inflammatory effects in a murine model [43]. The higher dose (100 mg/kg/day) corresponded to the AED of the maximum dose considered safe in humans (1000 mg/day) [38].

### 4.6. In Vivo Behavioral Assessments

#### 4.6.1. Assessment of Motor Activity/Forced Locomotion (Rotarod Test)

Animals were evaluated for motor activity using the rotarod test (IITC Life Science, Woodland Hills, CA, USA), with an acceleration speed of 16 rpm over a 300 s period. Prior to testing, animals underwent a seven-day habituation period, with sessions lasting three to five minutes. On the day of assessment, two trained examiners observed locomotion and assigned scores ranging from 1 to 5, where a score of 5 indicated normal paw use and a score of 1 indicated complete disuse. The final score for each animal was calculated as the arithmetic mean of the evaluators’ scores, thereby reducing individual bias [24,44,45].

#### 4.6.2. Spontaneous Locomotion (Walkway Gait Analysis)

Spontaneous locomotion analysis was employed to assess the animals’ locomotor function, using a glass walkway adapted at LEED/UFMA, in accordance with protocols previously described in the literature [46]. The apparatus measured 80 × 20 × 17 cm and was elevated 70 cm above the ground, allowing video recording with a fixed camera positioned on the floor at a distance of 70 cm from the walkway. Animals underwent an adaptation period prior to testing. During the experiment, displacement time and maximum speed achieved were recorded. Images were analyzed using Kinovea software (version 0.8.24), which provided precise measurements. Mean velocity (Vm) was calculated as the ratio between the distance traveled (Δs) and the time elapsed (Δt) (Vm = Δs/Δt), with final values obtained from the average of individual velocities within each group.

#### 4.6.3. Incapacitance Test/Hind Paw Weight Distribution (Weight-Bearing Test)

Spontaneous postural pain was assessed using the weight-bearing test, employing equipment from IITC Life Science (USA). This method allows quantification of changes in body weight distribution between the right hind paw, in which osteoarthritis was induced (OA+), and the left hind paw, which remained non-induced (OA−), with such variations serving as indicators of joint discomfort resulting from the lesion.

Animals were individually placed in a transparent restraint chamber coupled to a platform equipped with two independent load cells, capable of recording the weight exerted by each hind paw (measured in grams) without the need for immobilization. Each recording lasted five seconds, and three consecutive measurements were obtained per animal. The final value corresponded to the arithmetic mean of the three measurements.

Changes in weight distribution between the hind paws were expressed using the paw weight distribution index (PWDI), calculated according to the following formula [23,33,47]:

$$PWDI(\%)=\frac{PWDIA}{PWDIA+PWDIC}\times 100$$

where PWDI (%) is the percentage of the paw weight distribution index; PWDIA is the weight distribution index of the affected paw; and PWDIC is the weight distribution index of the contralateral paw.

#### 4.6.4. Quantification of Spontaneous Pain and Assessment of Mechanical Allodynia (Von Frey Test)

Mechanical allodynia was assessed using a digital Von Frey analgesimeter (model EFF 301W, Insight^®^, Ribeirão Preto, SP, Brazil). The device consists of a pressure transducer connected to a digital counter, allowing precise measurement (0.1 g resolution) of the force applied to the plantar surface up to a limit of 150 g. The stimulus was applied using a disposable polypropylene tip with a diameter of 0.5 mm attached to the transducer [48].

Animals were individually placed in transparent acrylic boxes (12 × 20 × 17 cm) with a metal mesh floor (5 mm^2^ grid), allowing access to the plantar surface of the paws. The boxes were positioned on an elevated platform with a mirror inclined at 45° and placed 25 cm below, facilitating visualization of the responses. After placement, animals were allowed to acclimate for 15 min before the start of the test [24,44,48].

The mechanical stimulus was applied gradually and continuously to the mid-plantar surface of the ipsilateral (affected) and contralateral paws until a paw withdrawal response occurred. Each paw was stimulated up to six times, and the three most consistent measurements were considered for calculation of the paw withdrawal nociceptive threshold (PWNT), expressed as the mean force (in grams) required to elicit withdrawal. Results were presented as a percentage of the PWNT, calculated according to the following formula [24,44]:

$$PWNT\left(\%\right)=\frac{PWNTA}{PWNTA+PWNTC}\times 100$$

where PWNT (%) is the percentage of the paw withdrawal nociceptive threshold; PWNTA is the paw withdrawal nociceptive threshold of the affected paw; and PWNTC is the paw withdrawal nociceptive threshold of the contralateral paw.

#### 4.6.5. Facial Expression of Pain (Rat Grimace Scale)

Facial expression of pain was assessed using the Rat Grimace Scale (RGS), a method developed to quantify spontaneous pain in rodents based on the observation of specific facial changes. Unlike tests that evaluate responses to thermal or mechanical stimuli, the RGS allows non-invasive and continuous assessment of animal discomfort, representing a sensitive and clinically relevant tool [29]. This scale was adapted from the Facial Action Coding System (FACS), originally used to assess pain in non-verbal patients, due to the similarity of pain-associated facial expressions between humans and rodents [29].

The assessment was based on four facial action units: orbital tightening, nose/cheek flattening, ear changes, and whisker change. Each parameter was scored on a scale from 0 to 2, corresponding to no pain (0), moderate pain (1), and severe pain (2). Analyses were performed by two independent evaluators, previously trained and blinded to the treatment groups, and the final score was determined as the mean of the values assigned by both observers [29].

### 4.7. Radiographic Evaluation

Radiographic evaluation of the knees was performed using a portable Diox^®^ device (Acteon, Rio de Janeiro, Brazil), equipped with digital exposure control, an LCD display, and automatic adjustment of exposure time and power, operating at fixed parameters of 60 kV and 4 mA. Images were acquired using a computerized system with a digital sensor (Micro Imagem, Indaiatuba, SP, Brazil), with exposure time, focus-to-film distance, and animal positioning kept constant. For image acquisition, rats were anesthetized with 2.5% isoflurane delivered via a face mask and positioned in dorsal and left lateral recumbency, and craniocaudal and laterolateral projections were obtained. Examinations were performed on experimental days D0, D7, D14, D21, and D28 in all groups.

Radiographs were evaluated in a blinded manner by an orthopedic specialist with expertise in osteoarthritis, using the Kellgren and Lawrence classification adapted for rodents. This classification is based on criteria such as joint space narrowing, osteophyte formation, subchondral sclerosis, and bone deformity, and ranges from 0 to 4, where 0 indicates a normal joint and 4 represents severe involvement, characterized by marked joint space narrowing, pronounced subchondral sclerosis, evident bone contour deformity, and the presence of large osteophytes. Each joint received a score, and results were expressed as the mean score per group [24,49,50].

### 4.8. Histological Evaluation of Inflammatory Parameters

Histological evaluation was performed at the Multiuser Histology Laboratory of the Post-Graduate Program in Health Sciences at the Federal University of Maranhão (LMH-PPGCS/UFMA). After euthanasia, the right knee joints were dissected and fixed in 10% buffered formalin (pH 7.2) for 48 h, then washed and stored in 70% ethanol for subsequent decalcification. Decalcification was carried out for approximately 15 days using EasyDesc Soft (EasyPath^®^, Indaiatuba, SP, Brazil—based on ethylenediaminetetraacetic acid [EDTA] and sodium and potassium tartrate), with weekly solution changes. After this period, samples were washed in running water and processed using standard histological procedures, including dehydration in an ascending alcohol series, clearing in xylene, and paraffin infiltration. The blocks were oriented to obtain horizontal sections, and 5 µm sections were cut using a semi-automatic microtome and stained with hematoxylin and eosin (H&E).

Histological slides were examined under a light microscope (Leica ICC50, Wetzlar, Germany) equipped with 10× eyepieces and 4× (500 mm), 10× (200 mm), and 40× (50 mm) objectives, and representative images were recorded as photomicrographs. For histological scoring, three representative sections per joint obtained from the central region of the femorotibial articulation were analyzed. The evaluation was performed in a blinded manner by a previously trained examiner, considering morphological changes in the articular cartilage, subchondral bone, and osteophyte formation. Cartilage degeneration was assessed using the semi-quantitative Osteoarthritis Research Society International (OARSI) scoring system adapted for rodents, which ranges from grades 0 to 6, where 0 indicates intact articular cartilage and surface, and 6 represents severe joint deformation and extensive cartilage damage [51]. Results were expressed as the mean histological score obtained for each experimental group.

### 4.9. Cytokine Quantification

Serum concentrations of IL-1β, IL-6, and IL-10 were determined by ELISA using commercial rat-specific kits (ELK Biotech, Wuhan, China). Blood samples were collected by abdominal aorta puncture at the time of euthanasia, and the obtained serum was stored at −80 °C until analysis. Samples were analyzed in triplicate, with standard curves prepared by serial dilutions according to the manufacturer’s instructions, and absorbance was measured at 450 nm [52].

Absorbance values were recorded in Microsoft Excel, and cytokine concentrations were calculated by interpolation from the standard curves, corrected for the dilution factor, and expressed as pg/mL (or normalized to total protein). Mean values were statistically analyzed using GraphPad Prism version 10.1.0.

### 4.10. Statistical Analysis

Data were analyzed using one-way analysis of variance (one-way ANOVA), followed by Tukey’s multiple comparison test. Variables influenced by two sources of variation (time × treatment) were evaluated using two-way repeated-measures ANOVA, also followed by Tukey’s post hoc test. Results were expressed as mean ± standard error of the mean (SEM), adopting *p* < 0.05 as the level of statistical significance. All analyses were performed using GraphPad Prism^®^ version 10.1.0.

## 5. Conclusions

The results of this study are promising and demonstrate that ambroxol exerts antinociceptive and anti-inflammatory effects in the MIA-induced osteoarthritis model, reducing pain, improving motor function, and attenuating articular degeneration, with efficacy comparable to that of meloxicam. The improvements observed in gait, weight distribution, and facial pain scores, together with the structural preservation detected by radiographic and histological analyses, reinforce the robustness of these findings.

Taken together, these results suggest that ambroxol has potential for therapeutic repositioning in osteoarthritis. However, additional studies are required to investigate its applicability in clinical trials and to further elucidate its molecular mechanisms of action.

## Data Availability

The original contributions presented in this study are included in the article. Further inquiries can be directed to the corresponding author.

## Abbreviations

The following abbreviations are used in this manuscript:

ABX	Ambroxol
AED	Animal equivalent dose
ANOVA	Analysis of variance
CEUA	Comissão de Ética no Uso de Animais (Institutional Animal Care and Use Committee)
CONCEA	National Council for the Control of Animal Experimentation (Brazil)
CTRL−	Negative control
D0	Day 0
D7	Day 7
D14	Day 14
D21	Day 21
D28	Day 28
ED-50	Effective dose 50%
EDTA	Ethylenediaminetetraacetic acid
ELISA	Enzyme-linked immunosorbent assay
FACS	Facial Action Coding System
H&E	Hematoxylin and eosin
IL-1β	Interleukin-1 beta
IL-6	Interleukin-6
IL-10	Interleukin-10
KL	Kellgren–Lawrence
LCD	Liquid crystal display
LEED	Experimental Laboratory for the Study of Pain
LMH-PPGCS	Multiuser Histology Laboratory—Graduate Program in Health Sciences
MIA	Monosodium iodoacetate
MLX	Meloxicam
Nav	Voltage-gated sodium channel
NF-κB	Nuclear Factor Kappa B
NSAIDs	Nonsteroidal anti-inflammatory drugs
OA	Osteoarthritis
OA+	Osteoarthritis-induced paw
OA−	Non-induced paw
OARSI	Osteoarthritis Research Society International
PWDI	Paw weight distribution index
PWDIA	Paw weight distribution index—affected paw
PWDIC	Paw weight distribution index—contralateral paw
PWNT	Paw withdrawal nociceptive threshold
PWNTA	Paw withdrawal nociceptive threshold—affected paw
PWNTC	Paw withdrawal nociceptive threshold—contralateral paw
RGS	Rat Grimace Scale
ROS	Reactive oxygen species
SEM	Standard error of the mean
TNF-α	Tumor necrosis factor alpha
UFMA	Federal University of Maranhão
Vm	Mean velocity
Δs	Distance traveled
Δt	Time elapsed
